# Nursing care experiences of people living with HIV/AIDS (PLWHA): A phenomenological study from the West of Iran

**DOI:** 10.1016/j.heliyon.2024.e40077

**Published:** 2024-11-01

**Authors:** Raheleh Rasad, Aliakbar Vaisi-Raygani, Alireza Abdi

**Affiliations:** a Nursing and midwifery school, student research committee, Kermanshah University of Medical Sciences, Kermanshah, Iran; b Nursing and midwifery school, Kermanshah University of Medical Sciences, Kermanshah, Iran

**Keywords:** HIV/AIDS, Patients, Nurses, Experiences, Care

## Abstract

**Introduction:**

Nursing care for people living with HIV/AIDS (PLWHA) can be challenging and is dependent on the context. However, there is a lack of information about nurses’ experiences in caring for PLWHA in the western region of Iran. Therefore, this study aimed to describe the experiences of nurses in caring for PLWHA in Kermanshah, Iran.

**Methods:**

In this qualitative descriptive phenomenological study, data were gathered via semi-structured, in-depth interviews. The sample comprised 15 nurses employed at hospitals affiliated with Kermanshah University of Medical Sciences, selected through purposive sampling. Colaizzi's seven-step method was employed to analyze the data, and MAXQDA 10 software was used for data management.

**Results:**

In this study, we obtained 579 codes and four main themes of "caring with fear", "Care ethics", "protective precautions" and "duality of feeling ".

**Conclusion:**

According to the results, nurses confront numerous difficulties, challenges, fears, and stress when dealing with PLWHA. Participants’ perceptions and practical experiences of AIDS patients enable patients to receive appropriate and high-quality care and provide valuable insights for nursing managers to enhance the preparedness of HIV ward nurses. To gain a comprehensive understanding of HIV nursing care, nurses require further education and awareness on this subject.

## 1 Introduction

HIV/AIDS remains a major public health challenge worldwide, especially in low- and middle-income countries [1]. Forty years ago, on June 5, 1981, the Centers for Disease Control's Morbidity and Mortality Weekly Report described five cases of Pneumocystis pneumonia in gay men [1]. This report heralded the HIV/AIDS pandemic, which has resulted in over 75 million HIV infections and 32 million deaths [2]. Approximately 2.1 million adolescents aged 10–19 years were living with HIV in 2016, with 90 % of these residing in low-resource settings (LRS) [3]. China has the lowest AIDS prevalence in the world, with only 0.1 % of adults affected [4]. The average cost of AIDS per month was $30.2, which accounted for 28.5 % of families' monthly incomes [5]. Despite significant progress in preventing HIV over the past decade, there is still fear among nurses when communicating with people living with HIV/AIDS (PLWHA). The fear of AIDS is known as one of the deadliest and most devastating public health crises because there is still no cure for the virus [6]. Regardless of this fact, nurses are expected to treat PLWHA the same as other patients. Some nurses still have non-acceptance of such patients and experience stress, fear, fatigue, and hopelessness when caring for PLWHA patients, even though they want to ensure that their patients receive the best care [6]. Walliams et al. found that nurses feared contracting AIDS because of perceived constant risk [3]. Previous studies have shown that some nurses and nursing students refuse to care for PLWHA due to fear of contagion. This negative attitude and unwillingness to provide care results in poorer quality of care [6,7]. Knowledge of AIDS and the needs of patients can reduce fear, anxiety, and stigma. Every nurse should know AIDS prevention, testing, treatment, and management to provide optimal care for those living with or exposed to the disease [8]. Nurses are expected to provide care, nutrition, comfort, and support to PLWHA regarding any challenges they may face in their work or personal lives [8]. Makhado and Davhana-Maselesele found that nurses do not have the knowledge or skills to fulfill their responsibilities in the field of AIDS care, leading to frustration at work [8].

Despite previous predictions that Islamic countries have low AIDS prevalence, the disease is a major public health crisis worldwide. Even in Islamic countries like Indonesia, AIDS epidemics have been spreading rapidly in recent years [6]. According to the Iranian Ministry of Health, AIDS remains a major threat to public health in Iran and is under review. Therefore, further research in this area is crucial [6]. Caring for people living with HIV/AIDS (PLWHA) can be a very stressful job for nurses. However, there is a lack of information about the experiences of nurses who care for PLWHA in Iran, especially in Kermanshah, a region with a higher prevalence of HIV. This study aimed to describe the experiences of nurses in caring for PLWHA in Kermanshah, Iran. The findings of this study can serve as a basis for developing strategies to improve the quality of nursing care provided for PLWHA.

### 1.1 Methods

In this qualitative study, a descriptive phenomenological approach was used. The purpose of this approach is to explore individuals' perceptions of their living experiences [9]. Phenomenology consists of two main approaches: interpretive and descriptive. The interpretive type, which focuses on interpreting the participants' life experiences, analyzes the elements reflecting the personal and subjective perspectives of individual experiences and attempts to report the participants' experiences, considering the researcher's view of the world. Likewise, interpretations are based on the researcher's perceptions, beliefs, expectations, and experiences [10]. In the descriptive phenomenological approach, individuals' perceived experiences are evaluated without the researcher's interpretation of meanings. This requires researchers to suspend their previous knowledge [11,12]. Given that the "nursing care experience of PLWHA" is an abstract concept and cannot be studied objectively, we should instead focus on the experiences of people who have provided this type of care [13,14]. Therefore, descriptive phenomenology was selected for this study.

### 1.2 Participants

The research population of this study consisted of nurses working in the internal, surgical, and infectious departments of educational hospitals at Kermanshah University of Medical Sciences. Fifteen nurses from these areas were recruited for the study using purposive sampling.

Three hospitals, according to the conditions of access to useful information about the research phenomenon (nursing care of PLWHA), were chosen among 19 government centers affiliated with Kermanshah University of Medical Sciences that had PLWHA in the internal, surgical, and infectious departments. We included nurses who had worked in medical, surgical, and infectious wards for a minimum of one year. They should have experience in caring for PLWHA in clinical settings and have consented to participate in the study. Additionally, they had at least a bachelor's degree in nursing, and they had gathered sufficient and comprehensive information about providing care for PLWHA (by asking the participants).

The sample size was determined through data saturation, which was identified when no new themes emerged from interviews and analysis [13]. In this study, data saturation occurred in the 13th interview, and two more interviews were conducted with two new participants to ensure that new themes did not emerge; thus, no new information was obtained at this stage.

### 1.3 Data collection

For data collection, the introduction letter was obtained from the Research Deputy of Kermanshah University of Medical Sciences, and the necessary permissions were obtained from the hospital centers where the research was conducted. The first researcher (RR) then went to the internal, surgical, and infectious departments. After introducing herself and presenting the title and objectives of the study and permission to the head of the departments, the profiles of the nurses and their work schedules were obtained. First, eligible nurses were identified to participate in the study. They were then provided with an explanation of the study's purpose and methodology, along with a guarantee of confidentiality and anonymity regarding their personal information. Finally, written informed consent was obtained from all participants. Then, an in-depth, semi-structured interview with each of the 15 participants who were selected using a purposive sampling method was implemented using open-ended questions such as "What are your experiences in caring for PLWHA?” What are the challenges of nursing care for PLWHA patients? What factors affect nursing care for PLWHA? The interview has been accepted as a valid method of data collection in qualitative research regardless of the type of study. In semi-structured interviews, the researcher starts interviewing with a set of open questions and spends considerable time searching for participating answers and encouraging them to provide details and explanations [13]. Simultaneously, probing questions such as, "May you explain more about this?" "Can you make this clearer?" or "May you clarify what you mean by an example?" were also used. Each participant received an interview, and at the end of each interview, the researcher reminded them that, if needed, they would be contacted by phone or in person about the contents to ensure an understanding of the interview findings. Each interview lasted between 30 and 60 min. The interviews were conducted following the agreement of the researcher and participants in the internal, surgical, and infectious departments during morning and evening shifts. The sampling process lasted approximately five months from April to August 2020. All interviews were recorded using a Samsung G7 cell phone.

The interviews and analysis were conducted by the study researchers; RR (MSc of nursing), AVR (an expert in qualitative research and nursing), and AA (an expert in qualitative research, critical care, and palliative nursing).

### 1.4 Data analysis

Data management was performed using MAXQDA-10 software. This software is used to organize and classify unstructured data such as interviews, articles, and audio and video files, during which it places similar notes or quotes in a group, and easy access to it makes it easy to compare with other groups [13]. All recorded interviews were analyzed using Colaizzi's seven-step approach. This approach is used to extract concepts related to people's living experiences and organize and analyze narrative data in descriptive phenomenology [15,16]. Thus, the following steps were performed: 1) At the end of each interview, the recorded statements of the participants were repeatedly listened to and their statements were written verbatim, and written interviews were studied several times. 2) By studying the interviews, the information related to the meaning and expressions related to the phenomenon was determined. 3) Then, a code that represented the meaning and fundamental part of the participant's thinking was extracted from each phrase 4) The researcher carefully studied the developed concepts(Codes) and sub-themes based on similarity. 5) The results were combined, and more general themes were created for a comprehensive description of the phenomenon under study, 6) a comprehensive description of the phenomenon under study was presented. In the final stage, the trustworthiness of data was addressed.

### 1.5 Trustworthiness

Data accuracy was based on Guba and Lincoln's approach [17], which consisted of four stages. A: To ensure the credibility of data, participants were asked to review the findings, comment on the accuracy of interpretations, and confirm the explanations (member check). The texts, codes, and classes were also examined by research colleagues (peer check). B: For dependability, the data collection method, data analysis, and contents were shared with external observers. Participants differed in demographic characteristics, including age, sex, workplace, and nursing experience, which increased the validity of the study. C: For conformability, the researcher extracted codes and themes from the participants' descriptions and tried to suspend the previous presuppositions. D: For transferability, the results of this study were provided to two nurses who were not participating in the study and their experiences were proportional to the results of the study. We also made a detailed description of the results [13].

### 1.6 Results

In this study, 15 nurses working in internal, surgical, and infectious departments participated in the study, of which 10 (66.6 %) were females, 8 (53.3 %) were married, and 14 (93.3 %) were BSCs (Table 1). Participants ranged in age from 25 to 52 years, and the average age and work experience were 36.33 and 12.73 years, respectively.

**Table 1:** Demographic characteristics of the participants.

Participant	Sex	Marital status	Age (years)	Workplace unit	Education	Work history in Infectious; Internal; Surgery departments (years)
P1	Male	Single	34	Infectious	BSc	9
P2	Male	Single	31	Infectious	BSc	9
P3	Female	Married	39	Infectious	BSc	16
P4	Female	Married	35	Infectious	BSc	11
P5	Female	Married	28	Infectious	BSc	4
P6	Male	Married	35	Infectious	BSc	11
P7	Female	Married	34	Internal	BSc	14
P8	Female	Single	40	Surgery	BSc	17
P9	Female	Single	25	Internal	BSc	3
P10	Female	Single	35	Surgery	BSc	10
P11	Female	Single	28	Surgery	BSc	6
P12	Female	Single	51	Surgery	BSc	26
P13	Female	Married	38	Surgery	BSc	11
P14	Male	Married	52	Surgery	MSc	28
P15	Male	Married	40	Surgery	BSc	16

**Fig. 1:**
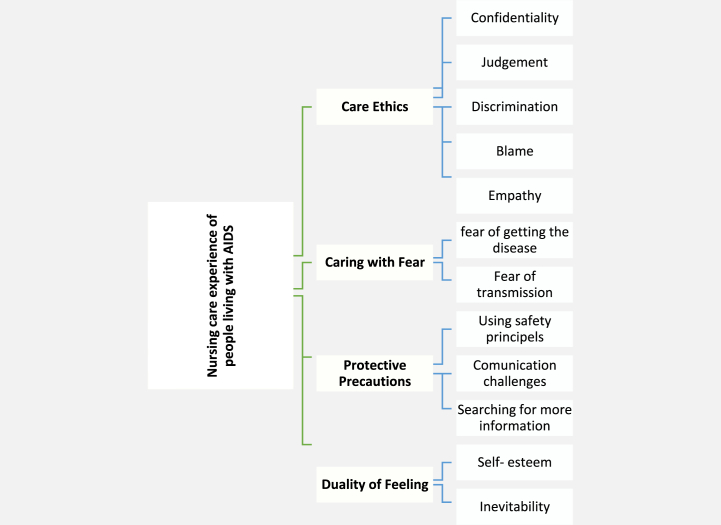
Coding tree of themes and sub-themes.

From the qualitative data analysis, four main themes "caring with fear”, “Care ethics”, “protective precautions”, “duality of feeling”, and 12 sub-themes appeared (Table 2, Fig. 1).

**Table 2:** The themes and subthemes of Nursing Care experiences of People Living with HIV/AIDS (PLWHA).

Themes	Subthemes	Examples of codes
**1. Caring with Fear**	Fear of getting the disease	Fear of disease, fear of infection, role of experience in reducing fear
	Fear of transmission	Needle stick worrisome, fear for family, fear of secretions
**2. Care Ethics**	Confidentiality	Importance of hiding the patient's information, being secret of data, prohibiting the revealing of information
	Judgment	Negative judgment about AIDS patients, judging patients, positive judgment
	Discrimination	Prejudice in care, discrimination between patients, lack of prejudice
	Blame	Place blame on the patient during care, fault for getting the disease, innocent patients
	Empathy	Walking in the patient's shoes, empathy with patients during care, helping helpless people
**3. Protective precautions**	using safety principles	Excessive attention to isolation, caution in care to prevent AIDS, compliance with the protocol
	Communication challenges	Having a distance and guarding against patients, AIDS as a barrier to communication, having an effective relationship
	Searching for more information	Searching for more data, curiosity on patients' events, elaborate check of records
**4- Duality of feeling**	Self-esteem	Sense of self-esteem, having merit for AIDS care, feeling happiness and satisfaction
	Inevitability	Force in caring for AIDS patients, compulsion to do the work, reluctance to do caring

## 2 Caring with fear

Participants stated that the concept of fear was intertwined in the care of a PLWHA. Most participants mentioned the fear of infection and subsequent distress, others were concerned about their family and those around them, and they experienced "fear of getting disease" and "fear of transmission" while caring for the PLWHA.

### 2.1 Fear of getting the disease

Participants mainly thought of PLWHA care as an important, dangerous, fearful, and health-threatening phenomenon. They described the fear of infection as fear of care, fear of needle sticking, and fear of exposure to patient discharge. Participant No. 2 who believed the fear of infection was higher at the beginning of nursing work, articulated:

"*The stress is normal at all, especially when facing the first HIV-positive patient, and (when) I encounter the HIV-positive patient for the first time, I felt stress that, for example, I was stressed about how to administer an IV (line) from him (the patient) to avoid becoming needle stick …* ". Most of the fears were during blood transfusion, inserting angiocaths, and contact with the patient's secretions. In this regard, participant No. 11 (28-year-old woman) stated:

"*Suddenly, (when) I want to get a vein (IV line), (I'm afraid) needles don't come into my hand, suddenly (patient) don't cough in my face, and all of a sudden everything or something doesn't fall in my eye. I was afraid of everything, I always said I'd get it (AIDS) now.”* Some nurses initially lacked the necessary knowledge or understanding and real attitude of AIDS, transmission methods, the exact percentage of probability of being infected with any needle stick, etc. In such situations, they experienced some worry and fear, but by receiving information, raising awareness, and creating a real picture of the phenomenon of HIV care, it was possible to prevent, mitigate, and reduce this fear. This meaning was well evident in the example statements of participant No. 15 (Mr. 40 years old) "*We need to know how the disease is transmitted when you know how the disease is transmitted, thus our care for the patient will be better."*

### 2.2 Fear of transmission

The concept of fear of transmission reflects that participants were afraid and stressed that they would become infected themselves and that their families would innocently contract AIDS. In this regard, participant No. 7 (34-year-old woman) explained: "*In the care of these patients, you may get an injury in spirit (psychotically injured), and you feel guilty and afraid that you will not get the disease (AIDS) and pass it on to your family* … ". Participant No. 13 (38-year-old woman) also stated: "*I would say that if I get married, my wife will also take it (AIDS), and I thought there would be no hope in life because there is no treatment, my life would be empty (of hope), andmy married life would fall apart*."

## 3 Care ethics

Based on the findings on the experiences of nurses who have cared for PLWHA, the communication process with these clients included a wide range of imagination, attitudes, judgments, empathy, and confidentiality, to degrading behavior, negative and judgmental attitudes, stigma and labeling, discrimination, and disclosure of patient secrets. In this regard, five sub-themes of "confidentiality", "judgment", "discrimination", "blame" and "empathy" appeared.

### 3.1 Confidentiality

Many participants stated that keeping patient information confidential is a patient's right and that it is a part of protecting patients' privacy. As Participant No. 6 (35 years old) stated: "*I, personally do not give the patient′s information even to his first-degree relatives unless the patient has agreed and I ask if you (the other people) are aware of his (the patient) illness. If I realize that he knows, I will talk to him about this more*." Participant No. 8 (a 40-year-old woman) also explained: "*A patient who is agitated and says that my mother-in-law does not know that I have AIDS. We avoid showing up (her disease) at all. We do not tell the patient's diagnosis at all*."

### 3.2 Judgment

Some participants stated that having a sense of responsibility and commitment to their profession makes them able to participate in nursing care regardless of why and how the patient is infected with AIDS. In this regard, participant No. 15 (Mr. 40 years old) stated: "*I am a nurse and the patient is infected for whatever reason, we may have to do our job and we should not pay attention to the cause. Most people with AIDS are not addicted, or if they have a problem, it is possible that a person with my personality has AIDS and (he/she) could not be judged."* However, sometimes nurses have negative and judgmental attitudes, e.g. highly contagious diseases, dirty people, delinquents, distancing themselves from people with AIDS, and avoiding caring for them. For example, participant number 11 (28 years woman) explained: "*At the beginning of my career, I judged the patient and said, Yeah, this young boy must have had an affair or was at a party, injected or (when) someone looked a little thin, (I thought) this was a drug addict who got (AIDS)* ".

### 3.3 Discrimination

Several participants underscored the significance of delivering healthcare services at a standardized level and upholding the rights of patients as clients in the care of PLWHA. In this regard, participant No. 15 (Mr. 40 years old) stated: "*To be honest, this patient is the same as the rest of the patients, so they should not be ashamed or nurses treat them in such a way that they think they have been rejected and should be treated like the rest of the patients.*" However, sometimes fear of occupational risk following continuous care of PLWHA avoids contact during care and induces discrimination in providing care services for these patients. Participant No. 12 (51-year-old woman, 26 years of experience) stated: "*Early on, when we went to the patient's head (patient's bedside), we didn't stay in her room long and tried to do our job earlier and come out*."

### 3.4 Blame

Participants stated that in caring for these patients, we should not blame all PLWHA for HIV/AIDS because some PLWHA were unintentionally infected and the patient should not be blamed for being infected with AIDS. Participant No. 11 (28-year-old woman) stated: "*But I said he (the patient) was sick, now every disease, I don't know how he got infected, he might have taken it (AIDS) in a way that has no fault*." However, some participants blamed the patient for the disease. As Participant No. 1 (Mr. 34) explained, "*I have sometimes been to blame patients and say that God is not satisfied with you for infecting yourself and (now) you're going to be trouble for us here*."

### 3.5 Empathy

Most participants stated that empathy and psychological support for PLWHA are among the most important aspects of nursing care. "*I've been working in this ward for 28 years and I always tell the kids (nurses/colleagues) to put yourself in his/her (AIDS patients) place for a moment and think they're your relatives, and we're never upset about what we're doing (for AIDS patients) if we look at the patient with this view*," said participant No. 14(Mr. 52 years).

Also, participant No. 8 (40 years woman, 17 years of work experience) explained: *"AIDS, because the patient, especially those who have been unwittingly infected, are under severe stress, have a helpless state, and I (as a) nurse, I have to put myself in the patient's place and mentally spend more energy to make my patient mentally stable otherwise his physical care is not very difficult for me like other patients, but mentally (is difficult). Like the mentally ill who are helpless and do not feel pleasant about the disease, they feel scared. As a nurse who has a responsibility on my shoulders in healthcare, I would change my patient mentally*."

## 4 Protective precautions

Nurses responsible for caring for PLWHA have experienced many psychological and emotional challenges. They used some precautions to protect themselves. In this regard, three sub-themes of "using safety principles", "communication challenges" and "searching for more information" were extracted.

### 4.1 Using safety principles

Participants commented that nurses should know the ways of AIDS transmission and prevent it by observing safety principles and providing routine nursing care to the PLWHA with more safety, accuracy, and mindfulness. In this regard, participant No. 12 (51-year-old woman) stated: "*We were doing the work of PLWHA at the end of the day (end of work shift) with more personal protective equipment than not going on the other patient's head and passing the disease on to them*."

Participant No. 7 explained, "*But our professors at the university explained very well that you should always be careful that any patient is HIV positive unless proven otherwise. Because of that, we always tried to approach the patient and do his job under health standards*."

### 4.2 Communication challenges

Some of the participants' statements during the interview indicated threatening behaviors experienced by PLWHA, and limited communication was one of the protective strategies that some nurses have used. Participant No. 11 (28-year-old woman) stated, “*I would have limited my connection to PLWHA, I took a position (an idiom means withdraw myself), and I would say that they got AIDS, and now I have to punish them, I didn't talk to them (PLWHA) much*." Moreover, she explained, " *Once, there was a homeless patient who was conscious and didn't have the money to do check out and he had AIDS and he said that if you don't let me go, I'll put a bloody needle on all of you (your body) and … And I was really afraid for a while to say no to hit me*".

However, a significant number of participants held the perspective that restricting communication could result in adverse psychological consequences. Conversely, they argued that fostering effective communication with the patient is pivotal in acknowledging the patient's humanity and cultivating a relationship founded on earning the patient's trust and confidence. As Participant No. 2 (Mr. 31) stated: "*By considering eye contact and talking to them (AIDS patients), they will unconsciously assure you how to communicate with you and tell you the history of the disease they already had. If their main complaint is easy to tell you, a discussion of confidence needs to arise*."

### 4.3 Searching for more information

Participants explained their experiences of curious behavior to learn more about how the patient is infected with AIDS. In this regard, Participant No. 7 (34-year-old woman) stated: "*If the patient has AIDS, I will definitely check the file (medical records) to see how he was infected. I looked at the patient's place of residence, even when I asked him how he contracted the disease and what problems had happened to him. And I'll ask him for an extra explanation."*

Participant No. 8 also stated: "*I asked the patient how he got infected, but most of the patients come to talk themselves (about their story of infection) because he (the patient) feels that he should give us (the reasons) and say how he got infected*."

## 5 The duality of feeling in care

Some participants stated that in the care of patients with AIDS, they felt excellent (had a special power) and satisfied with providing services to the PLWHA. In contrast, others had feelings of worthlessness, unlucky, and self-destructive. In this regard, two sub-concepts of "self-esteem" and "inevitability" appeared.

### 5.1 Self-esteem

The experience of self-esteem was reflected by some participants in PLWHA care as a sense of satisfaction and pleasure in helping the patient, feeling useful by providing scientific care, promoting self-confidence, and believing in the reward of this care in the sight of God. Participant No. 6 (Mr. 35 years old) stated: "*When you're working for an AIDS patient, you're doing something that other people don't do, and I'm going to take the place where other people are running away from it … It has two outcomes for me, one of which helps me with my self-esteem, which, is I do what other people don't do, and one that causes anxiety because I'm doing what others feel awful to do. And even his (the patient) family would not come to him (the AIDS patient)*".

### 5.2 Inevitability

Among the participants, only three did not feel satisfied with providing care to an AIDS patient, whereas they spoke of having experience, feeling worthless, self-destroying, and having to provide care to PLWHA.

“Participant No. 13 (Mr. 38 years old, 11 years of experience) stated, “But *we have to do it (care of AIDS patients), but with fear and with the help of colleagues … But now taking care of these patients is a good spiritual feeling because you're no longer satisfied with the patients' heads and you're not happy with your own and that you're doing it. God will see you and you will get something big for it, and you think this money you're bringing home is really legit because you're really playing with your own life (the life is in danger) and the nursing job is a sacred job*."

Participant No. 10 explained, "*The nurse is so desperate to take care of these patients. For example, something (AIDS patients) everyone is running away from, (the nurse) has to take care of him, even if there's a risk of infection, but he's got to do it*.” Participant No. 6 (Mr. 35 years old, 11 years of experience) also stated: "*Someone, who is caring for these patients, (this work) causes anxiety and self-desisting because I am doing what others are not trying to do and touch this patient and even his family does not come to him and reject him*."

## 6 Discussion

This study was conducted to explain the experience of nursing care for PLWHA from the perspective of nurses using a descriptive phenomenological approach. With this approach, the main themes that emerged from the analysis of participants’ statements in the present study include" caring with fear", " care ethics", "protective precautions", and "duality of feeling." Before providing care, nurses presented fear, stigma, and discrimination toward PLWHA, which may impact patient care [[18], [19], [20], [21], [22]]. Consistent with our findings, Reinius et al. [23] and Mahamboro et al. [24] suggest that healthcare providers often exhibit discrimination and prejudice against PLWHAs within communities and healthcare settings. In this study, caring with fear was one of the main themes of care associated with the fear of PLWHA. They experienced fears, stresses, and unpleasant and disturbing situations from the beginning without the intent to take care of PLWHA. The sub-concepts that played a role in the formation of the main concept of "caring with fear" can be referred to as "fear of disease" and "fear of transmission". According to our findings, this main concept has been demonstrated in other studies. Mashallahi et al. [25] also concluded that nurses in Ulmia are constantly afraid of being infected with viruses while caring for patients, which may make healthcare providers hesitant to engage in providing care to PLWHAs and, therefore, refuse to provide such care [26]. Tadzkiri et al. showed that nurses had a strong fear of contracting AIDS and reported the diverse impact of AIDS on healthcare providers' misunderstandings and fears [6]. The studies conducted by Qian et al. (2016) also showed that some nurses and nursing students do not want to care for AIDS patients due to the fear of contagious disease [4]. Bonacaro, Antonio et al. (2022) also reported that fear of AIDS and lack of knowledge about AIDS were identified as the main reasons for nursing students' reluctance to care for PLWHAs [7]. These fears may pose certain obstacles for both patients and nurses [18]. Hence, it is imperative to acknowledge and address these concerns preemptively, ensuring their mitigation before assigning a nurse to infection wards.

Another main theme of the findings of this study is " care ethics ", which includes five sub-concepts: "confidentiality", "discrimination", "judgment", "blame" and "empathy". According to our findings, Gutierrez et al. (2011) also reported that discrimination in the care and treatment of PLWHA is a challenging issue in healthcare facilities [19]. Healthcare providers and families with PLWHA should keep their interests in the patient, be his secret confidant, and not disclose his illness to others. However, according to the findings, even some healthcare personnel without sufficient knowledge of the problems of patients in the community unknowingly or curiously talk to their colleagues and others about their diagnosis and disclose it, which itself provides grounds for stigma and discrimination [29]. In addition, a study conducted by Saki et al. (2015) in Iran has shown that, like many other countries, people with AIDS often face discrimination when searching for and receiving healthcare services, which has serious consequences for their physical and psychosocial health [20]. Mahamboro et al. (2020) reported that stigma is a social structure that separates a person by a physical or social characteristic, resulting in negative social reactions such as discrimination and avoidance [24].

Our findings show that some nurses discriminate against PLWHA, and this discrimination is limited only to the early part of their experience of care. In confirming our findings, Monico et al. reported that discrimination in society is declining [6]. In line with our findings, in Tanzania, 49 % of health workers find themselves at risk of AIDS infection in the workplace and 13 % are reluctant to care for PLWHA [19]. Also, Tazkira et al. (2017) emphasized that most medical staff and nurses blame patients instead of addressing prevention issues. One male nurse explained: "When I heard the term HIV, I got angry and wanted to cry, "My God, it's not clear what he did, and now we have to make amends" [6]. Regarding care ethics issues of patients with AIDS, the compilation of specific guidelines/instructions tailored to their needs, along with the implementation of mandatory workshops, appears to be beneficial.

"Protective precautions" is another main concept obtained in this study. Nurses responsible for caring for PLWHA have experienced many psychological problems. They took some precautions to protect themselves, including "using safety principles," "communication challenges" and "searching for more information." According to our findings, Tazkira et al. (2017) also reported that the safety principle is another protective behavior reported by nurses in this study. One participant made the following comment: "When I saw the patient's test, I tried to apply the safety principles in such a way that the patient could not understand that I had a fear of developing the disease … " [6].

Limited communication was one of the protective strategies that some nurses used. Confirmation of part of our findings is seen in the Tazkira et al. study (2017), which reports that limited communication is one of the protective strategies that most nurses have used. A 45-year-old female nurse said "I'm not trying to communicate intimately and comfortably with a patient with AIDS … “He is not as friendly to me as some other patients" [6].

Some participants explained their experiences of curious behavior in order to learn more about how the patient is infected with AIDS. Tazkira et al. (2017) also reported that curiosity about exposure pathways was another aspect of caring for patients with AIDS. Nurses were curious to know how PLWHA were infected. Below is a quote from one of the female nurses: "I'm disappointed. I didn't know why? However, I wanted to know why the patient had AIDS. One day when I read his case, I realized that he was addicted … " [6]. It is evident that possessing clear information about patients, including details on transmission methods, as well as their psychological and physical symptoms, facilitates nurses in making informed decisions regarding patient care. Therefore, it is essential to include additional pertinent information in patient records and conduct comprehensive interviews with patients. Furthermore, the availability of personal protective equipment in infectious wards is imperative to ensure the safety of both patients and healthcare providers.

The concept of feeling duality is another main concept obtained in this research. The sub-themes formed in nurses who had experienced the care of PLWHA include "self-esteem" and " inevitability ". Some participants stated that they had experienced a great feeling of being happy and satisfied with the provision of services to these patients. Participants shared their feelings of "self-fulfillment," and they were proud of their work when the patient recovered from their sufferings, consistent with previous findings [26], while others had different experiences of providing care in a sense of worthlessness, bad luck, and self-destruction. The experience of feelings of worthlessness and self-destruction were among the new sub-concepts that were not found in any of the articles on the experience of nursing care for PLWHA.

HIV-focused educational interventions in clinical settings, such as experiential learning, working with PLWHA, and narrative photography, are recommended to facilitate nurses’ positive attitudes toward PLWHA [28]. Nurses also shared positive and effective practical experiences with HIV and AIDS services [28]. These experiences not only enable patients to receive fair and high-quality care but also better protect the occupational exposure of healthcare providers [28].

Another issue for student nurses is how to reduce potential occupational exposure. Contamination reduction considerations were the most important experiences and feelings of student nurses in caring for PLWHAs, which is congruent with the findings of previous studies)26). Compliance with universal precautions, such as wearing personal protective equipment (PPE), is a critical issue in caring, which protects nurses and allows them to provide quality care [27]. In our view, nurses who harbor negative feelings or attitudes regarding AIDS care should be reassigned to other hospital wards through rotations. This strategy mitigates the potential burden of burnout and its adverse effects on both patients and nurses.

### 6.1 Strengths and limitations

The nature of the research, geographical scope, question, and phenomenology method requires that the study be conducted only with the participation of several people who have experienced the care of PLWHA; therefore, the findings and results of this study, like other qualitative research, cannot be generalized to all other individuals, groups, and communities. However, we recruited participants from a wider range of hospitals to enrich the results, and the researcher had a long engagement with them to reach a deep understanding. The interviews explored the nurses’ understanding and experience of AIDS care without considering the experience of other stakeholders. Therefore, further research could explore the perspectives of nursing students, patients, and educators, which may provide additional insight into HIV-related care and education.

## 7 Conclusion

In this study, participants (nurses) mainly considered the care of PLWHA as an important, dangerous, fearful, and health-threatening phenomenon that was generally perceived as fear and anxiety. However, some of these issues were rooted in nurses' imagination, knowledge, and attitude. In a more general sense, based on the set of themes obtained from this study, caring for PLWHA is a set of fears, discriminations, judgments, blame, confidentiality, empathy, the use of safety principles, curiosity, communication challenges, and feelings that on the one hand have opened a dark inevitability and on the other hand a bright gateway to self-esteem. To provide care for PLWHAs, our findings can help nurse managers prepare young nurses to care for PLWHAs. To facilitate nurses’ positive attitudes toward PLWHA, recommendations such as decontamination education programs are identified in the present findings and can inform initiatives to address the challenges of nurses responding to the multiple needs of HIV patients. Future research may examine the applicability of the recommended approaches in nursing education and healthcare practice.

## CRediT authorship contribution statement

**Raheleh Rasad:** Writing – review & editing, Writing – original draft, Software, Methodology, Formal analysis, Data curation, Conceptualization. **Aliakbar Vaisi-Raygani:** Writing – review & editing, Writing – original draft, Validation, Supervision, Software, Methodology, Funding acquisition, Formal analysis, Conceptualization. **Alireza Abdi:** Writing – review & editing, Writing – original draft, Validation, Supervision, Software, Project administration, Methodology, Investigation, Formal analysis, Conceptualization.

## Declarations

**Ethics approval and consent to participate**: This study received approval from the Research Ethics Committee of Kermanshah University of Medical Sciences under the ID number: IR.KUMS.REC.1399.176. All participants provided written informed consent and were assured of the confidentiality and anonymity of their personal information.

**Data and code availability statement**: The data supporting the findings of this study are available upon request.

## Declaration of Competing Interest

The authors declare that they have no known competing financial interests or personal relationships that could have appeared to influence the work reported in this paper.
